# The possible place for psychedelics in pharmacotherapy of mental disorders

**DOI:** 10.1007/s43440-023-00550-9

**Published:** 2023-11-07

**Authors:** Adam Wojtas

**Affiliations:** Department of Pharmacology, Maj Institute of Pharmacology, Polish Academy of Sciences, Smętna 12, 31-343 Kraków, Poland

**Keywords:** Mood disorders, Fast-acting antidepressant drugs, Ketamine, LSD, Psilocybin, DMT, 5-MeO-DMT

## Abstract

Since its emergence in the 1960s, the serotonergic theory of depression bore fruit in the discovery of a plethora of antidepressant drugs affecting the lives of millions of patients. While crucial in the history of drug development, recent studies undermine the effectiveness of currently used antidepressant drugs in comparison to placebo, emphasizing the long time it takes to initiate the therapeutic response and numerous adverse effects. Thus, the scope of contemporary pharmacological research shifts from drugs affecting the serotonin system to rapid-acting antidepressant drugs. The prototypical representative of the aforementioned class is ketamine, an NMDA receptor antagonist capable of alleviating the symptoms of depression shortly after the drug administration. This discovery led to a paradigm shift, focusing on amino-acidic neurotransmitters and growth factors. Alas, the drug is not perfect, as its therapeutic effect diminishes circa 2 weeks after administration. Furthermore, it is not devoid of some severe side effects. However, there seems to be another, more efficient, and safer way to target the glutamatergic system. Hallucinogenic agonists of the 5-HT_2A_ receptor, commonly known as psychedelics, are nowadays being reconsidered in clinical practice, shedding their infamous 1970s stigma. More and more clinical studies prove their clinical efficacy and rapid onset after a single administration while bearing fewer side effects. This review focuses on the current state-of-the-art literature and most recent clinical studies concerning the use of psychedelic drugs in the treatment of mental disorders. Specifically, the antidepressant potential of LSD, psilocybin, DMT, and 5-MeO-DMT will be discussed, together with a brief summary of other possible applications.

## Introduction

Major depressive disorder is one of the most challenging social and medical problems of our time, affecting a significant part of the population. In fact, studies show that 5% of the world's adult population might suffer from this debilitating disorder [1], while up to 15% of the general population will experience depression at least once in their lifetime [2]. What is more, recent studies predict a significant increase in the number of affected individuals due to the COVID-19 pandemic [3].

The disease can exhibit a variety of heterogeneous symptoms, e.g., changes in weight and appetite (either increases or decreases), disturbances in sleep (either insomnia or hypersomnia), loss of energy, diminished self-esteem, cognitive impairments, and even recurring suicidal thoughts. Nevertheless, the two critical symptoms that need to manifest to diagnose depression are either prolonged depressed mood or anhedonia [4].

The etiology of major depressive disorder is poorly understood; however, a number of risk factors have been proposed, such as genetic predispositions, inflammation, disruptions in the gastrointestinal system (specifically, microbiota), fluctuations in concentrations of sex hormones, oxidative stress, and disrupted mitochondria functioning, but most significant one seems to be the exposition to traumatic or chronic stress [5–7].

Reaction to stress involves activating the hypothalamus–pituitary–adrenal (HPA) axis, which in response increases the release of glucocorticoids. While beneficial in the short term, excessive activation of the HPA axis and prolonged elevated levels of glucocorticoids can lead to disruptions in the functioning of various systems, including the central nervous system [8]. Animals exposed chronically to adrenal glucocorticoids display depressive phenotype while also exhibiting malfunctions in synaptic function, a decrease in the number of synapses, and neuronal atrophy and dysfunction in the cortical and hippocampal regions, symptoms parallel to that observed in depressed individuals [9–11]. In this review, the antidepressant potential of LSD, psilocybin, DMT, and 5-MeO-DMT will be discussed, together with a brief summary of other possible applications.

## Monoaminergic theory of depression and its implications

The monoaminergic theory of depression posits that imbalances in the levels of monoamine neurotransmitters in the brain, specifically serotonin, dopamine, and norepinephrine, contributes to the development of depression [12–15]. This paradigm originates in the two phenomena discovered in the 1950s, which are the impact of reserpine on the concentrations of aforementioned amines in the brain and the pharmacological activity of first antidepressant drugs. Moreover, later studies discovered abnormalities in the brain serotonin levels found in depressed individuals who committed suicide [16].

Reserpine, which is an alkaloid derived from *Rauwolfia serpentina*, was used in the 1950s as an antihypertensive [17]. However, it was observed to trigger depressive symptoms in some patients, which ceased upon the discontinuation of the treatment [18]. Additionally, similar effects were reproduced in animal models [19]. Reserpine was found to block the vesicular monoamine transporter, thereby causing depletion of brain monoamines such as serotonin and catecholamines [20, 21].

This evidence highlighted the possible role of serotonin, norepinephrine, and dopamine in depression.

Although developed as a treatment for tuberculosis, iproniazid was observed to elicit particular side effects that brought it to psychiatrists' attention, such as euphoria, psycho-stimulation, enhanced appetite, and improved sleep [22]. In a detailed clinical study, Loomer, Saunders, and Kline [23] administered iproniazid to depressed individuals—for several weeks, resulting in significant improvements in 70% of the participants. It was also found out that iproniazid is an inhibitor of the monoamine oxidase (MAO) enzyme [24]. This enzyme catalyzes the oxidative breakdown of biogenic amines like serotonin, dopamine, epinephrine, norepinephrine, and tyramine. There are two variants, MAO_A_ and MAO_B_, with different distributions in the body. Primarily, MAO_A_ is responsible for metabolizing serotonin, melatonin, norepinephrine, and adrenaline, while MAO_B_ oversees the breakdown of phenethylamine and benzylamine [25, 26]. When MAO is inhibited, the concentration of monoamine neurotransmitters increases in the presynaptic terminal, facilitating their release upon the arrival of action potentials.

Similarly to monoaminoxidase inhibitors, tricyclic antidepressant (TCAs) drugs were initially developed for another disorder, specifically as an improved version of the antipsychotic drug chlorpromazine [27]. It was later found that while lacking any antipsychotic activity (or even intensifying psychotic symptoms), imipramine administration led to significant improvement in depressed individuals [28]. TCAs exhibit a complex pharmacological profile, significantly impacting two reuptake transporters and three receptor proteins. They act by inhibiting norepinephrine and serotonin reuptake transporters, thus interrupting their uptake. Additionally, they block adrenergic α_1_ and α_2_ receptors, muscarinic receptors, and histamine H_1_ receptors [29–31]. The therapeutic efficacy of TCAs is hypothesized to primarily stem from the inhibition of norepinephrine and serotonin reuptake at the transporter proteins, which subsequently results in heightened concentrations of norepinephrine and serotonin within the synaptic cleft.

Based on the previous discoveries, the pharmaceutical corporation Eli Lilly initiated a more “goal-oriented” research in developing ligands that selectively inhibit serotonin reuptake at the corresponding transporters, thereby augmenting serotonin concentrations within the synaptic cleft to stimulate postsynaptic serotonin receptors more effectively. This resulted in the discovery of the selective serotonin reuptake inhibitor (SSRI), fluoxetine, in 1974 and describing its potential antidepressant properties’ medication [32]. In the succeeding year, Wong et al. demonstrated that fluoxetine manifested potent and selective serotonin reuptake inhibitory properties while exhibiting a relatively low affinity for the norepinephrine transporter [33]. This more selective profile and reduction in the possible side effects distinguished fluoxetine from the older antidepressant drugs, leading to it being approved by FDA in the 1987. Since then, a number of novel SSRIs have been developed and introduced to the pharmacotherapy of depressive disorders, making them the most commonly prescribed antidepressant drugs [34].

Yet, while providing significant relief for patients suffering from affective disorders, monoamine-based drugs are not without their flaws. First and most important is the fact that they exhibit a delayed onset, which usually requires several weeks of daily administration of the drug [35]. Combined with numerous adverse effects, this leads to poor compliance, with possible levels as low as 30% of the patients [36]. A large-scale meta-analysis by Cipriani et al. [35] revealed that while all antidepressants were more effective than placebo in adults with major depressive disorder, the effect sizes were generally small to moderate. Further contributing to this challenge is the relatively high placebo response rate in clinical trials for MDD, sometimes reaching up to 50%. Thus, while antidepressants statistically outperform placebo, the absolute difference in response rates often remains relatively small. What is more 30–50% of patients do not respond to available treatments [37], displaying the “treatment resistant” phenotype.

One possible explanation for the limited efficacy of antidepressants is that their mechanism of action involves elevating monoamine levels. However, people with depression do not necessarily exhibit lower levels of these neurotransmitters [38]. While antidepressant intake causes an immediate surge in monoamine levels, a delay in therapeutic effects is a common observation. It seems that the therapeutic benefits are mediated by subsequent changes in brain physiology, such as alterations in monoaminergic receptor levels and downstream cellular effects on enzyme cascades that affect metabotropic processes [39]. These changes then lead to modifications in the nuclear transcription of proteins such as brain-derived neurotrophic factor (BDNF) [5]. Essentially, current medications might be targeting an "incorrect" or at least an indirect pathway, which could compromise their effectiveness.

## The role of glutamate and mTOR pathway

It has been suggested that there may be dysregulation in glutamatergic neurotransmission in depressed individuals [40]. This is supported by research findings that have shown higher levels of serum and plasma glutamate in patients [41, 42], as well as a decrease in plasma glutamate levels after treatment with SSRIs [43]. Interestingly, the severity of depressive symptoms has been found to be correlated with plasma glutamate levels [44]. One possible explanation for the elevation of glutamate levels in MDD is the loss of glial cells that are responsible for regulating glutamate/glutamine cycling [45].

Trullas and Skolnick pioneered the notion that NMDA receptor pathways could play a role in the behavioral alterations arising from inescapable stress [46], thereby setting the foundation for the glutamatergic theory of depression. Their groundbreaking research showed that NMDA antagonists could alleviate depressive-like symptoms in animal models subjected to stress. This line of inquiry was further supported by follow-up studies, which validated the specific antidepressant effects of ketamine in animal models [47, 48]. This led to pioneering human studies [49, 50] which demonstrated the antidepressant properties ketamine exhibits in depressed individuals, an effect replicated in various later studies [51]. Furthermore, in contrast to monoaminergic antidepressants it has proven to be effective in individuals suffering from treatment-resistant depression [52].

The phenomenon behind the antidepressant action of ketamine involves a series of following steps [5, 53]. The drug preferentially inhibits NMDA receptors situated on γ-aminobutyric acid (GABA) interneurons [54], which are responsible for the inhibition of glutamatergic activity. Consequently, this leads to the downstream disinhibition of glutamatergic neurons and triggers a subsequent surge in glutamate, an event observed both in animal [55] and human [56] studies. The escalation in extracellular glutamate levels stimulates the activation of postsynaptic AMPA receptors, resulting in an influx of Ca^2+^, which in turn stimulates the release of BDNF. BDNF released into the synaptic cleft binds to tropomyosin receptor kinase B (TrkB), stimulating the Akt kinases, which activate the mTORC1 signaling pathways. The activation of the mTORC1 results in an increase in the synthesis of synaptic proteins which results in the restoration of synaptic plasticity and strength [57], which are disturbed in depressive individuals. What is more, inhibition of the mTORC1 pathway with rapamycin suppresses the antidepressant effects of ketamine treatment [58]. What is surprising, a recent study by Abdallah et al. found that co-administration of rapamycin actually prolongs the antidepressant effect of ketamine. While groundbreaking, the authors state that this effect has to be carefully replicated in further studies [59].

Despite its potential benefits, ketamine therapy has several limitations. One of the primary concerns is its dissociative effects, including hallucinations, derealization, and disorientation, which may be distressing for patients. These side effects are usually transient and diminish as the drug is eliminated from the body [60]. However, they can deter treatment and be dangerous if the patient is not adequately monitored. The administration of antidepressant therapy carries the potential hazard of an affective switch, specifically transitioning from a depressive to a manic state [61, 62]. Some case studies suggest that this might also happen when using ketamine as an antidepressant treatment [63]. Concerns have been raised about the hemodynamic responses related to ketamine use [64]. The term “Ketamine bladder” has been coined to describe a condition seen in individuals who misuse ketamine, characterized by symptoms such as hemorrhagic cystitis and painful urination [65]. Nausea is another commonly reported side effect, seen in approximately 30–40% of patients [66]. Furthermore, reports suggest that drowsiness and dizziness are experienced by 56.4% and 45.2% of patients, respectively [64].

Most importantly, the antidepressant effect of ketamine, while rapid, is also transient, usually lasting 1–2 weeks, which creates the need for repeated dosing to maintain the effect. Not much is known about the potential long-term effects of repeated administration of ketamine, and some studies suggest that there is a risk of neurotoxicity [67–69]. It has to be noted though that recent studies suggest that esketamine nasal application might be devoid of most of those adverse effects [70], but further studies are needed to confirm that.

All of the concerns stated above, while not denying the clinical significance of ketamine as a prototypical antidepressant drug, create a need for the development of rapid-acting antidepressant drugs that would maintain their properties for a more extended period while, if possible, exhibiting fewer adverse effects. A rising number of both preclinical and clinical studies suggest that psychedelics may share some similarities with ketamine, either by stimulating a surge of glutamate in the cortex [71, 72] or directly acting upon the TrkB receptor [73, 74], stimulating the mechanisms responsible for synaptic plasticity [75].

## The history and pharmacology behind psychedelics

Psychedelic substances have been used across different cultures throughout history. These substances, known for inducing profound changes in perception, mood, and cognitive processes, are often associated with therapeutic, religious, and recreational applications [76]. The earliest documented use of psychedelics dates back to prehistoric times, notably by indigenous cultures. The use of peyote, a small cactus containing the psychoactive compound mescaline, can be traced back over 5000 years in North America. Similarly, records of the use of psilocybin mushrooms, also known as “magic mushrooms,” have been discovered in ancient Saharan African and Central American cultures. These early uses were typically grounded in religious or shamanistic rituals, with the substances purportedly facilitating spiritual awakenings and mystical experiences [76, 77].

The modern era of psychedelic research started at the beginning of the twentieth century, initiated by studies regarding the hallucinogenic *Peyote* cactus. It was found that the active ingredient responsible for profound alterations in mood and perception was mescaline, and it was proposed for modeling mental disorders, specifically psychosis [78]. While inducing significant mind-altering effects, the effects of mescaline administration were qualitatively different, bearing little resemblance to psychosis [78]. The next chapter opened in 1938 with Albert Hoffman synthesizing lysergic acid diethylamide (LSD) and accidentally discovered its psychoactive properties in 1943 [79]. Due to its extreme potency, LSD quickly attracted the attention of psychiatrists and psychologists around the globe, becoming the most intensely researched psychedelic compound, with more than 1000 published articles by the end of the 1960s [78]. It was studied as a possible aid in psychotherapy, substance abuse disorders, anxiety, and mood disorders [80]. Psilocybin, the active compound of hallucinogenic mushrooms, was isolated also by Hoffman, in 1958 and studied, with possible application in neuroticism [81], autism, and schizophrenia [82] or facilitating psychotherapy [83]. Unfortunately, alongside medical use, some controversial figures like Timothy Leary advertised the recreational use of psychedelic drugs, quickly creating a widely spread association with counterculture and drug abuse [78]. This resulted in the passage of the “Controlled Substances Act” in 1970 and psychedelics being classified as “drugs with no currently accepted medical use and a high potential for abuse”. These circumstances made it difficult to continue research concerning psychedelic drugs and nearly all studies (with only a few exceptions) came to an abrupt end, followed by several decades of hiatus in psychedelic research. Nichols and Walter provide an excellent, comprehensive review concerning the history of psychedelic research.

Classical hallucinogens can be divided by their structure into two main categories: indoleamines, e.g., DMT (*N*,*N*-dimethyltryptamine) or LSD (Lysergic acid diethylamide), and phenethylamines, e.g., mescaline or DOI (2,5-Dimethoxy-4-iodoamphetamine) [72]. Both of them bear a resemblance to endogenous compounds—either serotonin (5-HT) or phenethylamine. While the former demonstrate affinity to several types of receptors, and nearly all 5-HT receptors [84], the latter bind mainly to the 5-HT_2_ receptor family [85, 86]. Hallucinogen use leads to quick development of tolerance to their effects [87, 88]. Moreover, indoleamines and phenylalkylamines exhibit a cross-tolerance phenomenon, which further suggests that they have a common mechanism of action [89]. Holistic data gathered from many studies indicate that hallucinogens exert their psychoactive effects by acting as agonists for cortical 5-HT_2A_ receptors [90–92]. The 5-HT_2A_ receptor belongs to the G-protein-coupled receptors (GPCRs). It is coupled with the Gq/11 protein, and its activation leads to phosphoinositide hydrolysis resulting in the formation of diacylglycerol and inositol triphosphate, leading to the mobilization of intracellular calcium and membrane depolarization [93]. What is more, the intensity of the psychedelic experience in humans is correlated with the occupancy of the 5-HT_2A_ receptor, mainly in the prefrontal cortex [94]. This activation of 5-HT_2A_ receptors in the prefrontal cortex launches a downstream cascade of changes in connectivity and alterations of blood flow across multiple regions of the brain, e.g., cingulate cortex, inferior parietal lobule, lateral temporal cortex, hippocampus, thalamus, amygdala, and claustrum [95–98]. Those structures are involved in cognition, emotional processing, sensory perception, or even self-recognition and theory of mind processes [77, 99].

Yet, despite their profound effect on neurotransmission, classic psychedelics seem to exhibit a safe pharmacological profile. Contrary to other classes of drugs they do not cause permanent harm, even in large doses, and are well tolerated when administered in a clinical environment [100–104]. What is more, both preclinical and clinical studies prove that, unlike other drugs, they are free of the abuse potential and their use does not lead to dependence [105].

On the other hand, the therapeutic use of psychedelics exhibits other challenges unknown to the other classes of drugs. The effects of the administration of a psychedelic compound are often ineffable, which makes it challenging to inform a patient of what their subjective experience could be like. What is more, this experience can be difficult and contradictory to their current worldview or values [106]. That is why, guidelines for therapists should be thoroughly researched and administrative regulations developed to minimize the ethical risks.

## Preclinical framework and clinical applications for psychedelic drugs

While the number of studies reporting beneficial effects of psychedelics both in healthy individuals and those suffering from mental disorders is gradually rising, the phenomenon behind those effects cannot be ascribed with certainty to their mechanism of action. Currently, we see two ways of explaining it; one could be called bottom–up, or “objective”, relying on neurobiological mechanisms, the other one top–down, or “subjective”, based on the qualitative values experienced under the influence of the drug.

The “objective” paradigm states that the administration of psychedelic compounds leads to drug-induced neuroplasticity, which restores the mechanisms of synaptic plasticity disturbed in depressive disorders associated with cortical atrophy and abnormal function [107]. This has been proven by demonstrating the psychedelic-induced plasticity in cell cultures. The observed changes included synaptogenesis, spinogenesis, and dendritogenesis [75, 108, 109]. Those findings were also replicated in brain slices, alongside more complex changes observed in the long-term potentiation in the cortex [75, 110, 111]. Finally, these effects were also observed in the in vivo studies for ketamine [112], DOI [113], psilocybin [114], and 5-MeO-DMT [115], with the effects of the latter two still significant a month after the treatment.

What is more, very recent studies discovered a possibility to mimic those effects by non-hallucinogenic analogs of psychedelic compounds [113, 116–119]. Yet, we have to remain careful, as these were only replicated in animal studies, and their antidepressant effect still has to be demonstrated in clinical studies. If that happens, it will be proven that subjective effects or the “trip” are not required for the therapeutic action.

The alternative approach focuses on psychedelics as facilitators of psychotherapy and enhancers of natural recovery. Studies concerning subjective effects invoked by psychedelics have numerously reported that the so-called “trip” can be one of the most meaningful and spiritual incidents one can experience [120–127], with beneficial effects lasting months after the drug administration, not only in depressed individuals but also in the healthy population.

What is interesting, even unpleasant and challenging experiences, so-called “bad trips” were reported to be beneficial [128]. Furthermore, the occurrence of mystical experience seems to be a predictable marker of successive treatment in depressed individuals [129].

### LSD

Due to its extreme potency and long duration of the acute, psychoactive effects (8–12 h) of its administration, LSD is rarely chosen for clinical studies. The first randomized, controlled trial (RCT) conducted according to modern scientific standards concerned the effectiveness of LSD in treating anxiety related to life-threatening diseases. Patients were treated either with 200 μg (treatment) or 20 μg (active placebo group) of LSD combined with psychotherapy. The result was a significant decrease in anxiety levels in the treatment group measured 2 months after the drug administration [130]. A follow-up study was conducted 12 months later, demonstrating a significant, long-term decrease in anxiety and beneficial changes to quality of life in the group treated with 200 μg of LSD [131].

During the first era of research in psychedelic drugs, LSD was seen as a promising agent in the treatment of alcohol dependency. While those studies were not as well designed as the contemporary research, a number of them were conducted as randomized-controlled trials, each one showing a significant decrease in alcohol consumption after a single dose of LSD in combination with a therapeutic program [132].

Apart from psychiatric disorders, LSD may also have applications in treating various kinds of pain. A study conducted in the Netherlands showed that low doses of LSD exhibit analgesic properties [133]. What is more, LSD seems to relieve pain associated with cluster headaches—acute, severe headaches of unknown etiology [134, 135], and clinical studies are needed to prove its effectiveness.

### Psilocybin

Psilocybin displays a significantly shorter duration of its action (4–6 h) than LSD, making it less difficult to study. With its favorable pharmacological properties, it has become recently the most intensely researched psychedelic compound. Quite similar to LSD, modern studies concerning psilocybin originated also in Switzerland, with the drug being used in research of psychotic disorders [136, 137]; which then evolved into basic studies describing the effects of psilocybin in healthy volunteers [138].

Furthermore, also akin to LSD, psilocybin was tested to examine possible beneficial effects on anxiety and distress associated with life-threatening diseases, specifically cancer. The first study was conducted in 2011; patients diagnosed with terminal cancer were administered psilocybin (0.2 mg/kg), and their primary measures were assessed 1 day prior, 1 day after, 2 weeks after, 1 month after, and 6 months after the drug administration. The measures of depression decreased by nearly 30% 1 month after psilocybin administration, and this effect was maintained at the 6-month follow-up, while the measures of anxiety decreased 1 month after the treatment and were maintained at the 3-month follow-up [139]. Another study conducted in 2016 examined the effects of psilocybin (0.3 mg/kg) administration on the depression and anxiety scores in cancer-diagnosed individuals and described significant improvement in both anxiety (58% of participants) and depression (83% of participants) measured 7 weeks after the treatment) [140].

Psilocybin was also examined as a treatment for addictions. When combined with Cognitive-Behavioral Therapy (CBT) for cessation of smoking, 80% of participants maintained abstinence at the 6-month follow-up, 67% at the 12-month follow-up, and 75% at the 2.5-year follow-up [141, 142]. Moreover, Bogenschutz et al. [143] demonstrated that psilocybin treatment acutely decreased alcohol consumption after the dosage and the effect was still significant at a 36-week follow-up. A randomized-controlled trial concerning the use of psilocybin in cocaine-use disorder is currently on its way (NCT02037126), while research concerning the use of psilocybin in opioid addiction is also being planned.

The most advanced research involving the therapeutical use of psilocybin refers to its potential antidepressant properties. Carhart-Harris and colleagues demonstrated that double administration of psilocybin (first with a low dose of 10 mg and a higher dose of 25 mg 7 days later) produced significant effects in 67% of patients with treatment-resistant depression in the first week after the treatment, in a 3-month follow-up, 47% of treated individuals stayed in remission [144]. What is more, this effect was persistent as measured in a 6-month follow-up [145]. The randomized-controlled trial (NCT03181529) conducted at Johns Hopkins demonstrated that two psilocybin sessions (20 mg/70 kg) and (30 mg/70 kg) combined with psychotherapy induced a significant improvement in depressive symptoms in 71% of participants in week 1 and week 4 of the study, while 58% of participants in week 1 and 54% of participants in week 4 were in remission [146]. What is more, Gakyusan et al. [147] reported long-lasting antidepressant effects of psilocybin administration, with significant response to treatment maintained in 75% of participants and a remission rate of 58% in a 12-month follow-up.

While psychedelics might offer a novel way of treating depressive disorders, especially that of the treatment-resistant kind, careful studies should assess their effectiveness in comparison to ketamine and classical antidepressant drugs. Clinical trial NCT03429075 led by Carhart-Harris demonstrated no significant differences between the antidepressant effect induced by two doses of psilocybin (25 mg) separated with a 3-week interval and a daily, 6-week treatment with escitalopram [148]. The follow-up analysis by Barba et al. [149] revealed reduced rumination and thought suppression in the psilocybin group, while only reduced rumination in the escitalopram group. This may be due to the different mechanisms of action of the drugs, psilocybin acting through the 5-HT_2A_ receptor, is associated with active coping with stress, while escitalopram acting indirectly through the 5-HT_1A_ receptor is associated with promoting resilience to stress in depression [150]. To further investigate if the activation of the 5-HT2A receptor is necessary for the antidepressant effect, the clinical trial NCT05710237 will investigate if co-administration of risperidone (5-HT2A receptor antagonist) undermines this effect. What is more, the rapid-acting antidepressant properties of psilocybin are currently being investigated in comparison to ketamine to evaluate its’ potential superiority (clinical trial no. NCT05383313).

One study suggested that psilocybin is safe and well tolerated in individuals with obsessive–compulsive disorder (OCD) and is associated with the reduction of OCD symptoms [151]. Those findings encouraged a group at Yale University to conduct clinical trials investigating psilocybin (0.25 mg/kg) effects on OCD symptomatology (NCT03356483).

### DMT and ayahuasca

Ayahuasca, a psychoactive brew made of plant material containing inhibitors of MAO and *N*,*N*-Dimethyltryptamine, has also gained significant attention from researchers in the twenty-first century. First, its physiologic effects were examined among the members of Santo Daime church, one of the few organizations permitted to use it during religious rituals [152, 153]. Ayahuasca ingestion increased blood flow in the insula, cingulate cortex, medial prefrontal cortex, and amygdala and a significant decrease in activity in the Default Mode Network (DMN), a neurocircuitry associated among others with depressive disorders [153, 154]. These studies are in line with the results obtained from other psychedelics, like psilocybin or LSD, which also diminish DMN activity [155, 156] and may suggest a hypothesis for their antidepressant properties.

The studies mentioned above-provided premises to test the antidepressant properties of ayahuasca. The first results came from Osorio and colleagues [157], as they reported that a single ayahuasca administration could significantly reduce the symptoms of depression up to 82%, 1, 7, and 21 days after the drug administration while not triggering any episodes of mania. The same group replicated their study a year later in a larger sample group, demonstrating a rapid and significant drop in depressive symptoms. What is more, an additional SPECT study demonstrated increases in blood flow in the nucleus accumbens and insula, regions linked with mood and emotion processing. Ayahuasca did not induce any life-threatening adverse effects and was generally well tolerated; the only observed adverse effect was vomiting [158]. Furthermore, ayahuasca was tested in a randomized-controlled trial (NCT02914769) as a treatment for treatment-resistant depression. For 64% of participants, there was a significant decrease in depressive symptoms, while 36% of them were in complete remission as measured 7 days after the drug administration [159]. While all of those results seem promising, follow-up studies at much more distant time points should be conducted to assess if those effects are only short term or if the antidepressant effect is maintained.

While not orally active without monoamine oxidase inhibitors, *N*,*N*-DMT can also be administered alone via inhalation or injection. The psychoactive effects are much more rapid and intense, with a duration of about 1/2–1 h, depending on the road of administration [160]. Anecdotal reports coming from users suggested that it may also exhibit therapeutic properties [161, 162]. A recently completed clinical trial (NCT04673383) reported impressive results in depressed individuals with intramuscularly administered DMT. At a 3-month follow-up, 57% of participants were in complete remission. This is especially interesting due to two factors—the short-term psychoactive effect induced by DMT compared to other psychedelics and a robust antidepressant effect maintained over a long period. Currently, DMT is being investigated in individuals undergoing SSRI therapy, but for whom it is not entirely alleviating their symptoms (NCT05553691).

### 5-MeO-DMT

Very recent studies point to the 5-MeO-DMT as a possible rapid-acting antidepressant drug. Epidemiological studies reported that the drug decreases symptoms of PTSD (79%), depression (77%), and anxiety (69%) in diagnosed individuals [161], and the potency of the effect correlated with the intensity of mystical experience. A clinical study (NCT04698603) by Reckweg and colleagues [163, 164] assessed the safety 5-MeO-DMT application via inhalation route in individuals with treatment-resistant depression, reporting the adverse effect being moderate and resolving spontaneously, with no life-threatening adverse effects. These results prove that the drug may be well tolerated and safe to use. What is more, it invoked a very rapid antidepressant effect, with all of the patients having their symptoms significantly reduced and 87% of them being in total remission in a 1-week follow-up [164]. What is more, it is currently considered for bipolar disorder (NCT05839509) and post-partum depression (NCT05804708). Similarly to clinical trials concerning the rapid antidepressant effects of ayahuasca, larger sample studies with more distant follow-ups should be conducted to address the effectiveness of 5-MeO-DMT in depressed individuals.

## Conclusions

The current renaissance in psychedelic drug research provides us with an alternative approach to the treatment of mental disorders. There are premises that psychedelics could help us with mood disorders, anxiety-related disorders, and addictions, especially in patients not responding sufficiently to currently used drugs and therapeutic protocols. While the preliminary results seem promising, more studies like Carhart-Harris and colleagues [148] comparing psychedelic treatment and classically used drugs have to be conducted to judge the superiority of either of the existing paradigms. What is more, there is some evidence [165] that, sometimes, the combination of those approaches may give us synergistic results, improving both resilience to stress and active copying mechanisms while reducing the adverse effects associated with psychedelics [165], this possibility is currently investigated in the clinical trial no. NCT05594667.

## Abbreviations

5-HT: Serotonin
5-MeO-DMT: 5-Methoxy-*N*,*N*-dimethyltryptamine
Akt: Protein kinase b
AMPA: α-Amino-3-hydroxy-5-methyl-4-isoxazolepropionic acid
BDNF: Brain-derived neurotrophic factor
CBT: Cognitive-behavioral therapy
DOI: 2,5-Dimethoxy-4-iodoamphetamine
DMN: Default-mode network
DMT: *N,N*-Dimethyltryptamine
GABA: γ-Aminobutyric acid
GPCR: G-Protein-coupled receptor
HPA: Hypothalamus–pituitary–adrenal
LSD: Lysergic acid diethylamide
MAO: Monoamine oxidase
MDD: Major depressive disorder
mTOR: Mammalian target of rapamycin
mTORC1: Mammalian target of rapamycin complex 1
NMDA: *N*-Methyl-d-aspartic acid
OCD: Obsessive–compulsive disorder
RCT: Randomized controlled trial
SPECT: Single photon emission computed tomography
SSRI: Selective serotonin reuptake inhibitors
TCA: Tricyclic antidepressant drugs
TrkB: Tropomyosin receptor kinase B

## Declarations

### Data availability

Data sharing is not applicable to this article as no datasets were generated or analyzed during the current study.
